# Blockade of α4 integrins reduces leukocyte–endothelial interactions in cerebral vessels and improves memory in a mouse model of Alzheimer’s disease

**DOI:** 10.1038/s41598-019-48538-x

**Published:** 2019-08-19

**Authors:** Enrica Pietronigro, Elena Zenaro, Vittorina Della Bianca, Silvia Dusi, Eleonora Terrabuio, Giulia Iannoto, Anna Slanzi, Somayehsadat Ghasemi, Rajasekar Nagarajan, Gennj Piacentino, Gabriele Tosadori, Barbara Rossi, Gabriela Constantin

**Affiliations:** 1 Department of Medicine, University of Verona, 37134 Verona, Italy; 2 The Center for Biomedical Computing (CBMC), University of Verona, 37134 Verona, Italy

**Keywords:** Chronic inflammation, Alzheimer's disease

## Abstract

Alzheimer’s disease (AD) is a neurodegenerative disorder characterized by cognitive decline associated with the deposition of amyloid-β (Aβ) plaques, hyperphosphorylation of tau protein, and neuronal loss. Vascular inflammation and leukocyte trafficking may contribute to AD pathogenesis, and a better understanding of these inflammation mechanisms could therefore facilitate the development of new AD therapies. Here we show that T cells extravasate in the proximity of cerebral VCAM-1^+^ vessels in 3xTg-AD transgenic mice, which develop both Aβ and tau pathologies. The counter-ligand of VCAM-1 – α4β1 integrin, also known as very late antigen-4 (VLA-4) – was more abundant on circulating CD4^+^ T cells and was also expressed by a significant proportion of blood CD8^+^ T cells and neutrophils in AD mice. Intravital microscopy of the brain microcirculation revealed that α4 integrins control leukocyte–endothelial interactions in AD mice. Therapeutic targeting of VLA-4 using antibodies that specifically block α4 integrins improved the memory of 3xTg-AD mice compared to an isotype control. These antibodies also reduced neuropathological hallmarks of AD, including microgliosis, Aβ load and tau hyperphosphorylation. Our results demonstrate that α4 integrin-dependent leukocyte trafficking promotes cognitive impairment and AD neuropathology, suggesting that the blockade of α4 integrins may offer a new therapeutic strategy in AD.

## Introduction

Alzheimer’s disease (AD) is the most common form of dementia, affecting ~6% of the global population aged >65 years^1^. Its clinical symptoms include progressive memory loss, impairment of daily activities, deterioration of language, visuospatial deficits, and mood changes^2^. Both the familial and sporadic forms of AD share common neuropathological features: the progressive accumulation of β-amyloid (Aβ) in brain parenchyma and vasculature, neurofibrillary tangles (NFTs) containing hyperphosphorylated tau protein, synaptic dysfunction and neuronal loss. Current therapies are symptomatic and do not alter the course of AD, therefore new drugs are urgently needed to slow the progression of this disease^2^.

Chronic low-grade inflammation is thought to play a key role in the development of AD neuropathology. Previous studies of inflammation in AD have focused mainly on the activation of microglia^3^. However, several recent studies have shown that a dysfunctional blood–brain barrier associated with vascular inflammation and leukocyte migration into the brain contribute to the pathogenesis of AD^3–5^. In particular, we and others have shown that circulating leukocytes adhere to the endothelium in cerebral vessels and migrate into the brain parenchyma in AD patients and in transgenic animals with AD-like disease^3–7^. Our recent data show that neutrophils (the most abundant leukocytes in the human circulation) adhere in cerebral vessels and migrate into the AD brain. They also migrate into the brain parenchyma of 3xTg-AD and 5xFAD mouse models of AD at the onset of memory deficit, secreting IL-17 and producing neutrophil extracellular traps (NETs) that may harm endothelial and neural cells^7^. CD4^+^ and CD8^+^ T cells can also adhere in cerebral blood vessels and migrate into the parenchyma of AD patients, with most T cells infiltrating the hippocampus and other limbic structures, the most severely affected areas in AD^6,8–12^.

Leukocyte migration from the circulation through the inflamed vascular wall is a multistep process mediated by adhesion molecules and chemoattractants. It involves a succession of events including: (1) tethering (capture) and rolling, which are mediated by adhesion molecules from the selectin, mucin and integrin families and the immunoglobulin (Ig) superfamily; (2) chemoattractant-triggered integrin activation via leukocyte G-protein-coupled receptors; (3) firm adhesion (arrest) mediated by activated integrins and their counter-ligands from the Ig superfamily; (4) crawling mediated by β1 or β2 integrins and their ligands; and (5) transmigration (diapedesis)^13,14^. Integrins are a large group of heterodimeric adhesion molecules composed of α and β subunits. The most important β_1_ integrin expressed on leukocytes is α_4_β_1_ integrin (CD49d/CD29), also known as very late antigen-4 (VLA-4). Integrin α_4_β_1_ plays a pivotal role during T cell trafficking in the chronically inflamed central nervous system (CNS) in multiple sclerosis and its animal model, experimental autoimmune encephalomyelitis^13,15^. However, VLA-4 also contributes to the migration of monocytes, eosinophils and neutrophils during inflammatory responses^16–19^. CD49d (the α4 chain of VLA-4) can be expressed with either of two β chains, namely β1 (CD29) or β7, to form VLA-4 (integrin α_4_β_1_) or integrin α_4_β_7_, respectively. Integrin α_4_β_1_ principally binds to VCAM-1 on the vascular endothelium and mediates leukocyte trafficking to sites of inflammation, whereas interactions between integrin α_4_β_7_ and its vascular ligand MAdCAM-1 (mucosal addressin cell adhesion molecule-1) plays a more specific role in naive T cell homing in the high endothelial venules of Peyer’s patches^13,14^. Targeting the α4 chain of VLA-4 has a therapeutic effect in patients with multiple sclerosis, a chronic inflammatory disease of the CNS, and in Chron’s disease, an autoimmune disease of the intestine, demonstrating that blocking key adhesion mechanisms controlling leukocyte migration can efficiently mitigate chronic inflammation in human diseases.

Leukocyte migration in the CNS has been investigated almost exclusively in the context of stroke and multiple sclerosis. However, the molecular mechanisms controlling leukocyte trafficking in the AD brain are largely unknown. Here we investigated the role of α_4_β_1_ integrin in leukocyte–endothelial interactions in the cerebral microcirculation of 3xTg-AD mice, which develop both amyloid and tau pathologies. We also used an antibody to block the α4 chain of VLA-4 to determine its therapeutic effect on cognitive functions and the neuropathological hallmarks of AD. We found that VLA-4 contributes to the inflammatory response in 3xTg-AD mice and could therefore be targeted for AD therapy.

## Results

### T cells accumulate near cerebral VCAM-1^+^ blood vessels in 3xTg-AD mice

**Figure 1:**
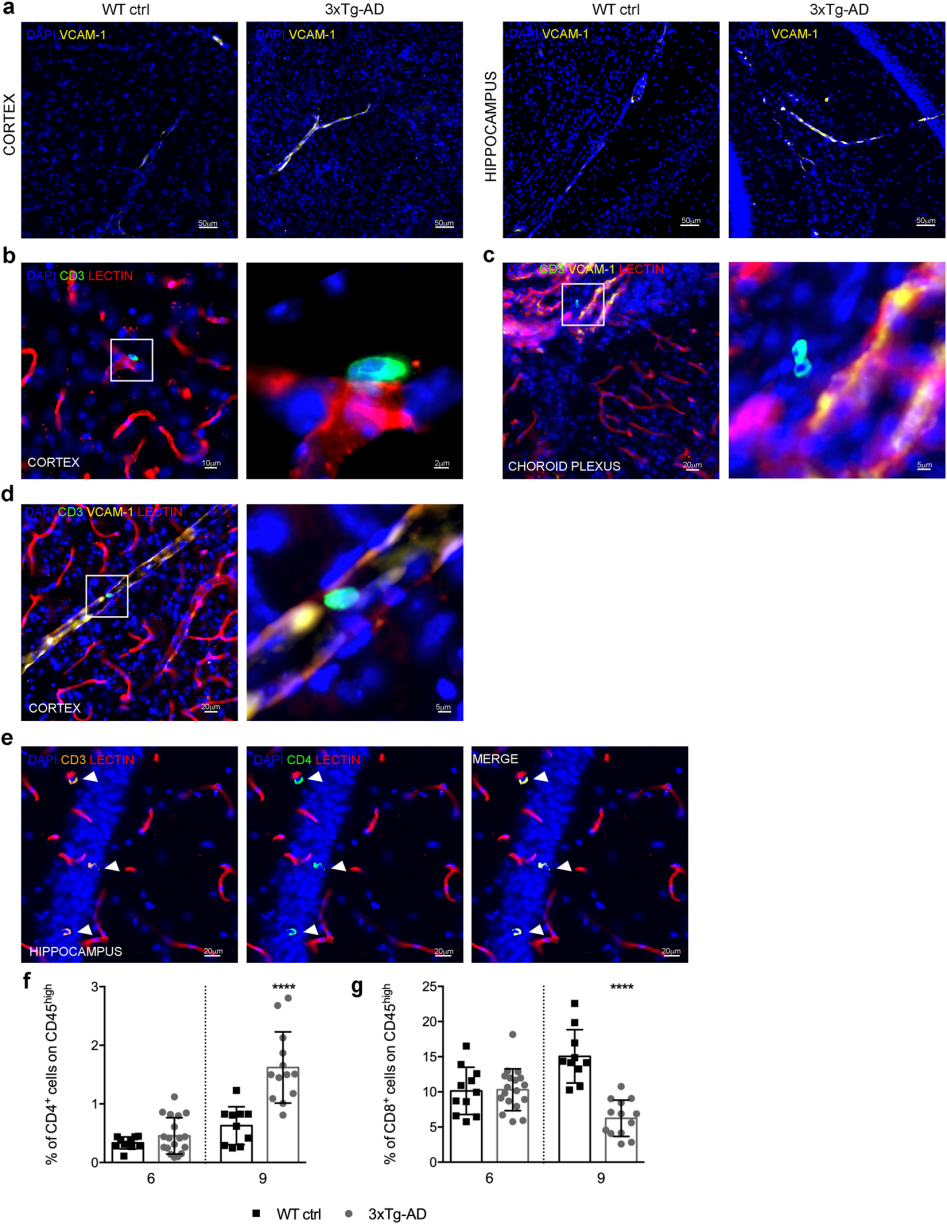
Presence of T cells near VCAM-1^+^ vessels in the brains of 3xTg-AD mice. (**a**) Confocal microscopy showing representative images of VCAM-1 expression in sections of the cortex and hippocampus in 3xTg-AD mice compared to wild-type control mice (WT ctrl) at 6 months of age. (**b**) Extravasation of a CD3^+^ T cell from a cortical vessel stained with lectin in a 3xTg-AD mouse (magnification of the quadrant is shown in the right-hand image). (**c**) Presence of an extravasated CD3^+^ T cell near a VCAM-1^+^ vessel in the choroid plexus (magnification of the quadrant is shown in the right-hand image). (**d**) Adherent T cell inside a VCAM-1^+^ vessel in the cortex (magnification of the quadrant is shown in the right-hand image). Nuclei are stained with DAPI (blue), blood vessels are marked with Texas Red tomato lectin (red), VCAM-1 is stained with an antibody conjugated to Alexa Fluor 647 (yellow), and T cells are stained with Alexa Fluor 488 using an anti-CD3 monoclonal antibody (green). (**e**) Infiltrating CD3^+^ (orange)/CD4^+^ T cells (green) highlighted with arrowheads in the hippocampus of 3xTg-AD mice at 6 months of age. Quantitative flow-cytometry analysis of infiltrating CD4^+^ (**f**) and CD8^+^ T cells (**g**) in 3xTg-AD mice (black bars) compared to wild-type control mice (WT ctrl) (grey bars) at 6 and 9 months of age. Data are expressed as mean ± SD. (****P < 0.0001; Mann-Whitney U-test). At 6 months: n = 11 mice (5 F, 6 M) for the WT ctrl group and n = 18 mice (9 F, 9 M) for the 3xTg-AD group. At 9 months: n = 10 mice (5 F, 5 M) for the WT ctrl group and n = 13 mice (7 F, 6 M) for the 3xTg-AD group.

The expression of endothelial adhesion molecules is necessary for the recruitment of circulating leukocytes to sites of inflammation. Among the vascular adhesion molecules, VCAM-1 has a pivotal role during chronic inflammatory responses by recruiting circulating T cells. Our previous studies have shown that endothelial adhesion molecules, including VCAM-1, are detected in the CNS vessels of 6-month-old 3xTg-AD mice, which express transgenes corresponding to three mutant human proteins: PS1 (M146V), βAPP (Swedish) and tau (P301L)^20^. To determine whether VCAM-1 expression is associated with the recruitment of circulating leukocytes, we stained brain sections prepared from 3xTg-AD mice for VCAM-1 and T cells. This confirmed higher vascular VCAM-1 expression in the cerebral cortex and hippocampus of 3xTg-AD mice at 6 months of age compared to wild-type control mice (Fig. 1a and data not shown). Furthermore, we observed the presence of extravascular migrating T cells, with some cells near VCAM-1^+^ vessels in the cerebral cortex and choroid plexi of 3xTg-AD mice, suggesting that vascular inflammation and VCAM-1 expression may contribute to T cell recruitment to the AD brain (Fig. 1b,c). We also observed the adhesion of CD3^+^ cells inside VCAM-1^+^ cortical blood vessels (Fig. 1d). Staining with an anti-CD4 antibody confirmed that the CD3^+^ T cells infiltrating the brains of 3xTg-AD mice also express CD4 (Fig. 1e). Taken together, these data suggest that VCAM-1 is significantly more abundant in the brain vessels of 3xTg-AD mice and may mediate T cell migration into the CNS in these animals. To assess T cell infiltration in the brains of wild-type and 3xTg-AD mice in more detail, we conducted flow cytometry experiments on brain homogenates. We observed an increase in the abundance of CD4^+^ T cells at 6 and 9 months of age in the brains of 3xTg-AD mice compared to wild-type animals, with the results reaching statistical significance at the later time point (Fig. 1f; Supplementary Fig. 1). However, the number of CD8^+^ T cells migrating in the brain was similar in wild-type and 3xTg-AD mice at 6 months, but was significantly lower in 3xTg-AD mice than wild-type animals at 9 months (Fig. 1g; Supplementary Fig. 1), suggesting that the role of CD8^+^ T cells may diminish over time in 3xTg-AD mice.

### Differential expression of α4 integrin on peripheral leukocytes in 3xTg-AD mice

**Figure 2:**
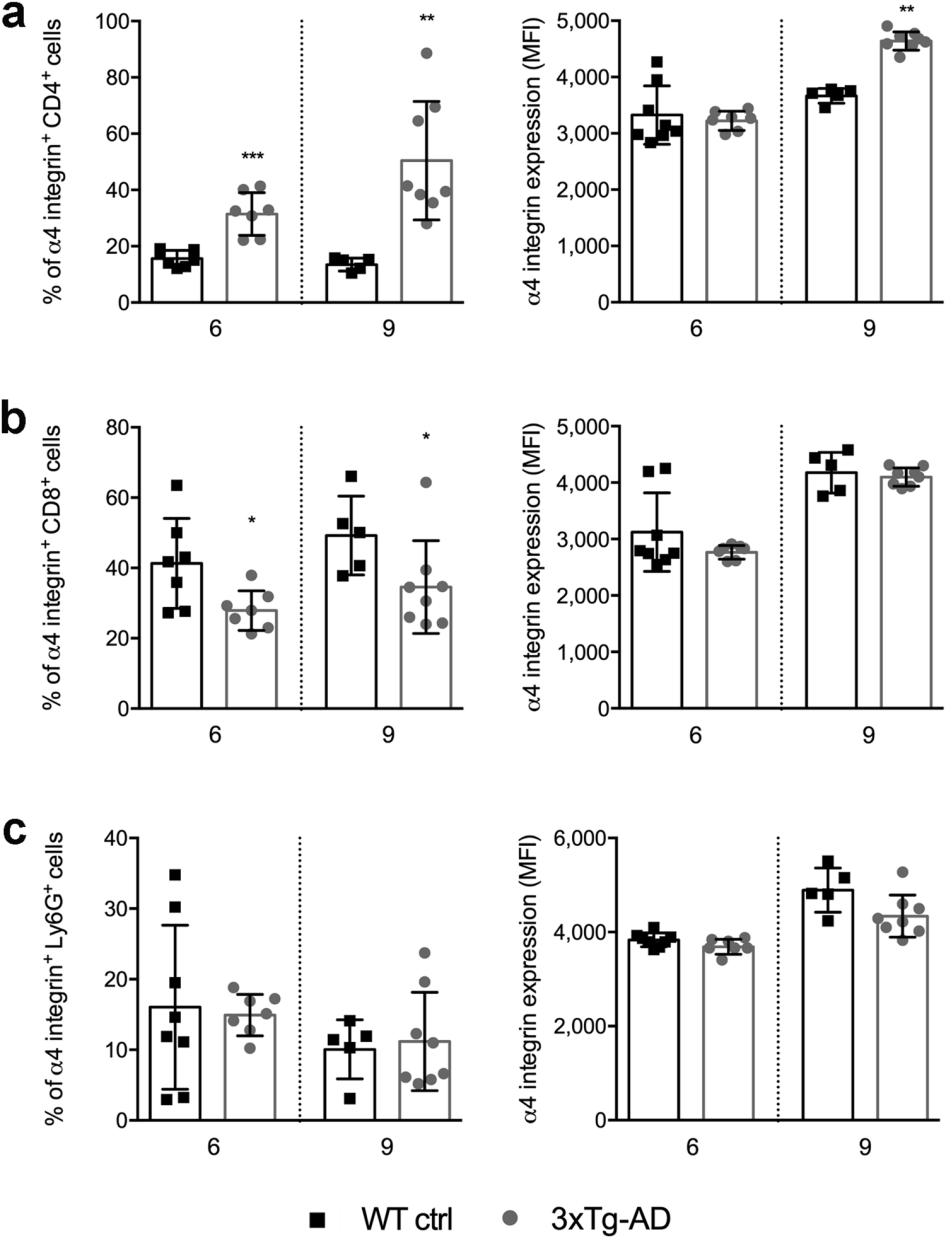
The expression of α4 integrin on circulating leukocytes in 3xTg-AD mice. Quantitative flow-cytometry analysis of peripheral CD4^+^ T cells (**a**), CD8^+^ T cells (**b**) and Ly6G^+^ cells (**c**) in 3xTg-AD mice (white bars) compared to wild-type control mice (WT ctrl) (black bars) at 6 and 9 months of age. For all cell populations, the graphs show the proportion of cells expressing α4 integrin and the mean fluorescence intensity (MFI) of normalized α4 integrin expression. Error bars represent SD (*P < 0.05, **P < 0.01, ***P < 0.001; Mann-Whitney *U*-test). At 6 months: n = 8 mice (3 F, 5 M) for the WT ctrl group; n = 7 mice (3 F, 4 M) for the 3xTg-AD group. At 9 months: n = 5 mice (2 F, 3 M) for the WT ctrl group and n = 8 mice (4 F, 4 M) for the 3xTg-AD group.

VCAM-1 mainly functions as a trafficking receptor for VLA-4 (α4β1 integrin) so we next checked the expression of the α4 integrin chain (CD49d) on blood leukocytes in 3xTg-AD mice compared to wild-type controls at 6 and 9 months of age by flow cytometry. Our data revealed a progressive age-dependent increase in the proportion of CD4^+^ T cells expressing α4 integrin and the mean fluorescence intensity (MFI) representing α4 expression increased in AD mice compared to controls (Fig. 2a; Supplementary Fig. 1). CD49d was also expressed on CD8^+^ T cells, although the proportion of those cells was slightly lower in AD mice compared to wild-type controls (Fig. 2b; Supplementary Fig. 1), suggesting these cells may rely on different trafficking mechanisms compared to CD4^+^ T cells in AD mice, a finding previously observed in other contexts of brain inflammation^21^. CD49d was also expressed on a significant proportion of circulating neutrophils (Ly6G^+^ cells), although there was no difference between AD mice and wild-type controls in this regard (Fig. 2c; Supplementary Fig. 1). We also observed no differences in the mean fluorescence intensity (MFI) of CD8^+^ cells and neutrophils between the groups. Together, these data suggest that CD49d expression could be used as a marker for the progressive activation of peripheral CD4^+^ T cells in AD mice and that α4 integrins may contribute to leukocyte recruitment in the CNS of 3xTg-AD mice.

### Blocking α4 integrins reduces leukocyte–endothelial interactions in brain venules of AD mice

**Figure 3:**
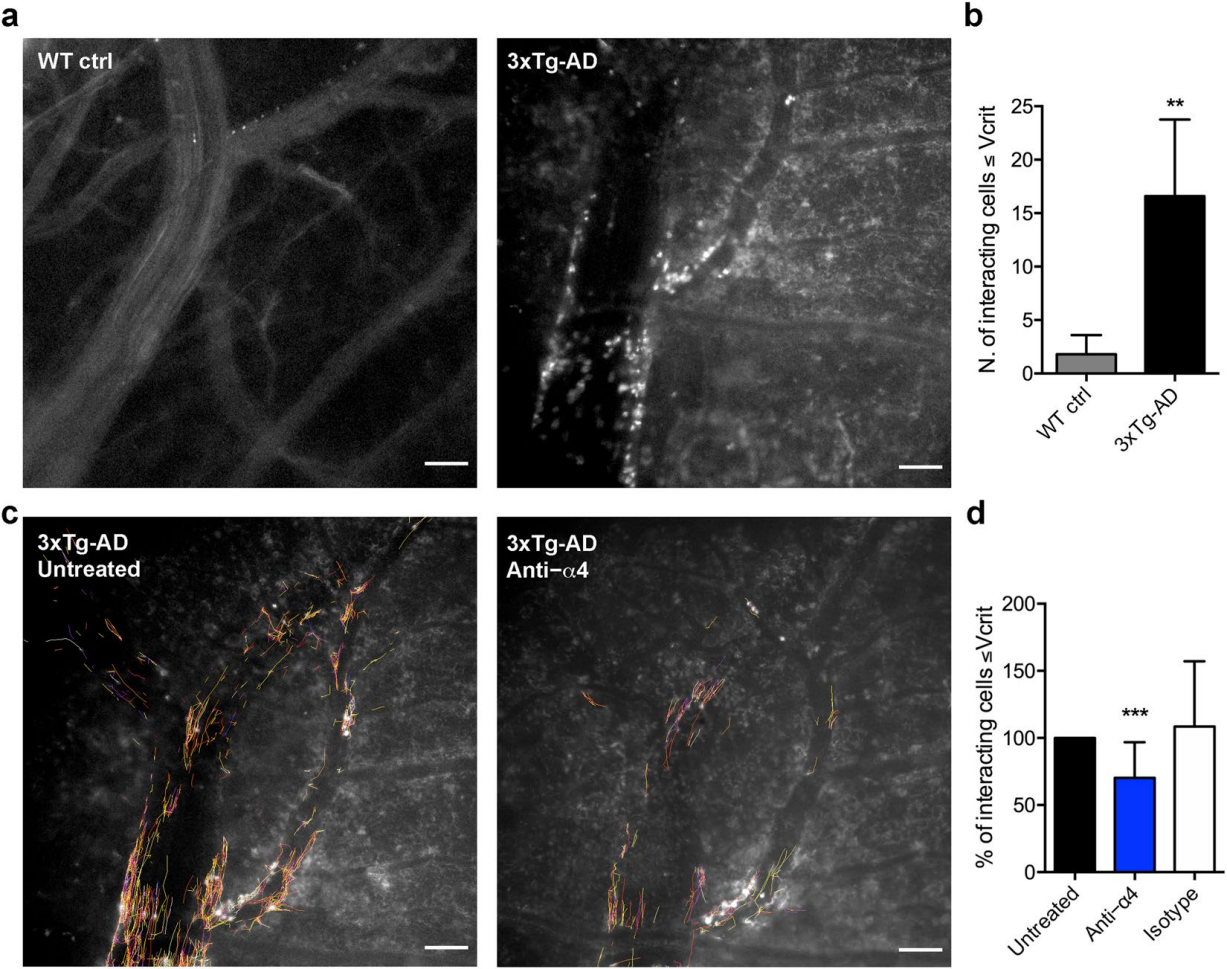
Increased leukocyte–endothelial interactions in the brains of AD mice and the effect of α4 integrin blockade. (**a**) Representative images from EIVM experiments using a digital camera to record endogenous leukocytes labeled with Rhodamine 6 G, and automatic cell tracking in cerebral blood vessels of wild-type control (WT ctrl) and 3xTg-AD mice at 6 months of age. Interacting cells were automatically tracked in the field of acquisition based on their centroid fluorescence intensity. Scale bar = 100 µm. (**b**) Automatic Imaris analysis of endogenous leukocytes shows rare interacting cells in wild-type control (WT ctrl) mice compared to 3xTg-AD mice (**P < 0.01, Mann-Whitney *U*-test). (**c**) Representative images of leukocyte tracks in cerebral blood vessels of 3xTg-AD mice before (left panel, Untreated) and after (right panel, Anti-α4) i.v. injection into the lateral tail vein with a blocking antibody against α4 integrin. Scale bar = 100 µm. (**d**) Automatic Imaris analysis of leukocyte tracks before (Untreated) and after 30 min of treatment with the α4 integrin-specific blocking antibody shows a significant reduction in the percentage of interacting cells in the treated mice compared to the before-treatment control, set at 100% (***P < 0.001, Mann-Whitney *U*-test), whereas no effect was observed with an isotype control antibody. Bars show the percentage of interacting cells in the same venule over the course of 1 min. At least three vessels per mouse were analyzed and three animals were tested for each condition. Data are expressed as means ± SD (n = 3 mice (F) for all experimental conditions).

T cells and neutrophils have previously been shown to accumulate in the brain in several transgenic models of AD-like disease^4^. To demonstrate that α4 integrins mediate the adhesion of leukocytes in the cerebral microcirculation of AD mice, we next performed epifluorescence intravital microscopy (EIVM) experiments using two types of recording and analysis systems to compare the cerebral microcirculation of 3xTg-AD mice and wild-type control animals at 6 months of age. We first used a digital camera system (2.5 frames/s) to directly observe and record endogenous leukocyte adhesion within cerebral microvessels directly. Automatic high-throughput data analysis using Imaris software revealed a much greater number of rolling interactions in 3xTg-AD mice compared to wild-type controls (Fig. 3a), clearly demonstrating that the brain endothelium in AD mice is activated to allow the adhesion of circulating leukocytes (Fig. 3b). Following the acquisition of baseline interactions in 3xTg-AD mice, we administered a single intravascular (i.v.) of a blocking antibody specific for α4 integrin and recorded endogenous leukocyte interactions 30 min later (Fig. 3c). Notably, automatic analysis demonstrated that blocking α4 integrin significantly reduced the percentage of interacting cells (traveling at velocities below V_crit_) in 3xTg-AD mice (***P < 0.001) (Fig. 3d). No significant effect was observed following the administration of an isotype control antibody (data not shown).

**Figure 4:**
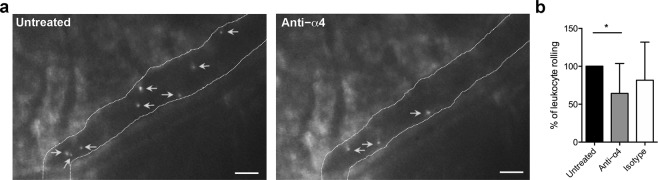
Blocking α4 integrin reduces endogenous leukocyte rolling in the cerebral vessels of 3xTg-AD mice. (**a**) Representative EIVM images using an analog camera to record the rolling of endogenous leukocytes labeled with Rhodamine 6 G in the cerebral blood vessels of 3xTg-AD mice before (left panel, Untreated) and after (right panel, Anti-α4) i.v. injection into the lateral tail vein with a blocking antibody against α4 integrin. (**b**) Manual analysis of leukocyte rolling before (Untreated) and after 30 min of treatment with a blocking antibody against α4 integrin or an isotype control antibody shows a reduction in the percentage of rolling cells after treatment compared to the control acquisition, set at 100% (*P < 0.05, one-way ANOVA with Tukey’s multiple comparison test). The isotype control antibody showed no significant effect on rolling. Bars show the percentage of interacting cells in the same venule over the course of 1 min. At least three vessels per mouse were analysed and three animals were tested for each condition. Data are expressed as mean ± SD (n = 3 mice (F) for all experimental conditions).

To confirm the results obtained by automatic EIVM, we repeated the analysis using a more sensitive analog camera (25 frames/s) allowing the more accurate detection of adhesive interactions, followed by the manual analysis of the digitized movies (Fig. 4a). This showed that blocking α4 integrins significantly reduced endogenous leukocyte rolling in 3xTg-AD mice (*P < 0.05), whereas no significant effect was observed following treatment with an isotype control antibody (Fig. 4b). As expected, fewer firmly adhering cells were observed after the blockade, but the number of the arresting cells was very low and these data were not plotted or shown. Overall, our findings support the presence of vascular inflammation in AD mice and demonstrate that α4 integrins contribute to leukocyte–vascular interactions in cerebral vessels in mice with AD-like disease.

### Therapeutic blockade of α4 integrin improves memory in 3xTg-AD mice

**Figure 5:**
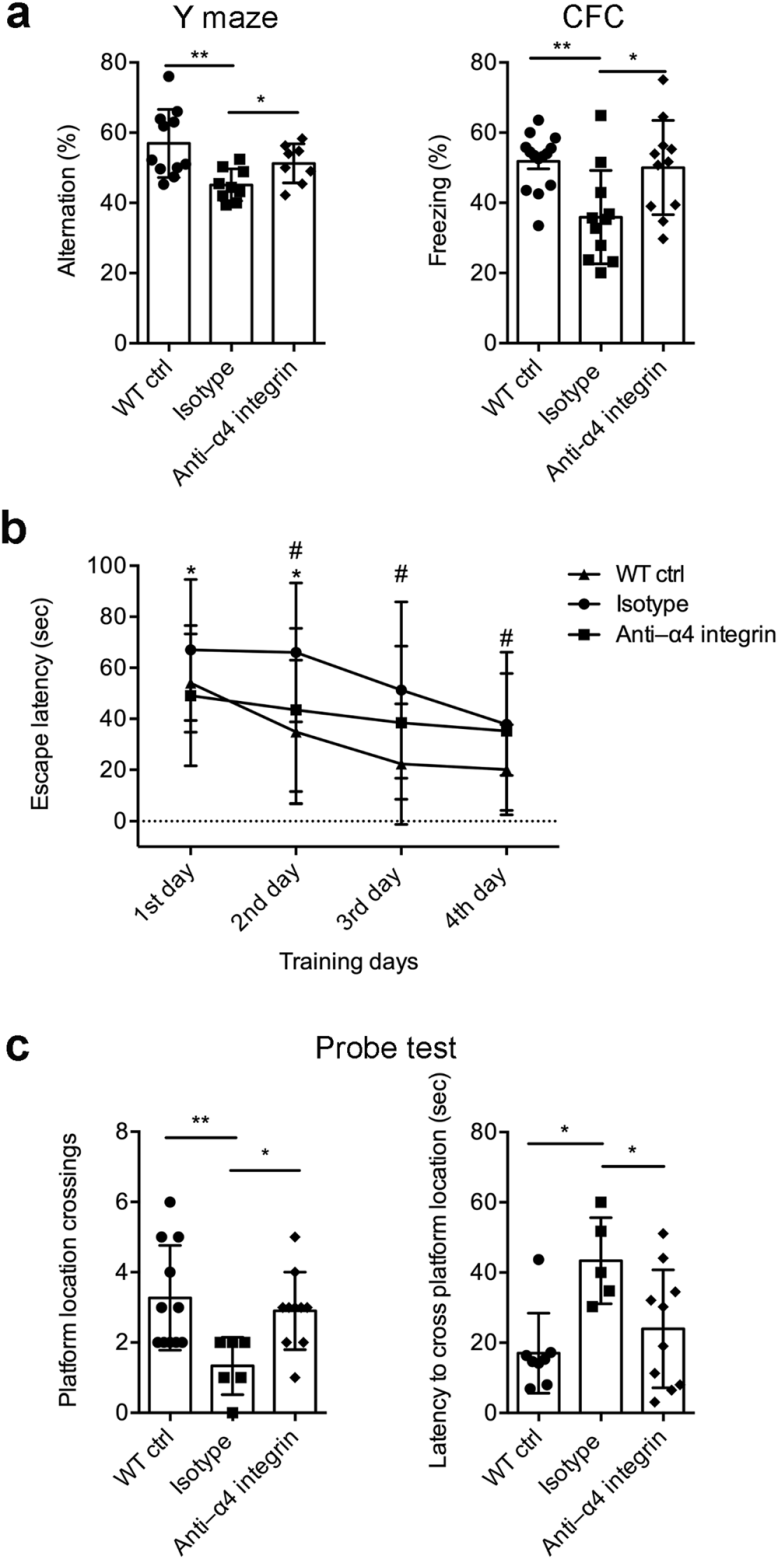
Blocking α4 integrin improves cognitive functions in 3xTg-AD mice. Groups of 3xTg-AD mice were treated with a blocking antibody specific for α4 integrin (Anti-α4 integrin) or an isotype control antibody (Isotype) for 4 weeks starting at 6 months of age. Wild-type age-matched control mice were treated with endotoxin-free PBS. (**a**) Results showing the percentage of alternation in the Y-maze test (left) and the percentage of freezing in the contextual fear-conditioning (CFC) test (right) Y-maze, n = 11 mice (5 F, 6 M) for the WT ctrl group, n = 9 mice (4 F, 5 M) for the isotype group, and n = 8 mice (4 F, 4 M) for the anti-α4 integrin group. For CFC, n = 14 mice (7 F, 7 M) for the WT ctrl group, n = 11 mice (5 F, 6 M) for the isotype group, and n = 11 mice (6 F, 5 M) for the anti-α4 integrin group (*P < 0.05, **P < 0.01; Mann-Whitney *U*-test). (**b**,**c**) Morris water maze test. (**b**) The escape latency to reach the hidden platform during training period (two-way ANOVA between groups: **P < 0 01 and between training days; **P < 0.01; Multiple t tests within the same day *P < 0.05 wild-type control (WT ctrl) versus isotype control; ^#^*P* < 0.05 isotype control versus treatment group). (**c**) Number of platform location crossings and latency to cross platform location (*P < 0.05, **P < 0.01; Mann-Whitney *U*-test). Values represent mean ± SD of the data obtained from a representative experiment with n = 11 mice (5 F, 6 M) for the WT ctrl group, n = 8 mice (4 F, 4 M) for the isotype group, and n = 10 mice (5 F, 5 M) for the anti-α4 integrin group.

To confirm that VLA-4 influences the pathogenesis of AD-related neuro-inflammation, we treated 6-month-old 3xTg-AD mice, which already show cognitive deficits^20^, with the α4 integrin-blocking antibody. After treatment for 4 weeks, the mice were allowed to rest for another 4 weeks and were then tested for cognitive functions at 8 months of age. In the Y-maze spontaneous alternation test and contextual fear-conditioning test, the therapeutic blockade in 3xTg-AD mice restored memory compared to mice treated with an isotype control antibody (Fig. 5a). Cognitive functions in the treated mice were comparable to those in age-matched wild-type littermates in both tests, highlighting the efficacy of this early therapeutic intervention in AD mice. We observed no significant differences between males and females in the behavioral tests (data not shown). Blocking α4 integrin in wild-type mice had no effect on cognition, further supporting a role for leukocyte adhesion in cognitive dysfunction mediated by neuroinflammation in AD mice.

Furthermore, 3xTg-AD mice treated with the therapeutic blockade also showed a memory improvement in the Morris water maze (MWM) assessment, i.e. a significant reduction of escape latency during the training period compared to mice treated with a control antibody (Fig. 5b). There were no significant differences in swimming speed between the treatment group and controls (wild-type 0.185 ± 0.038 m/s; isotype 0.213 ± 0.045 m/s; anti-α4 0.227 ± 0.055 m/s, mean ± SD). In addition, the therapeutic blockade achieved a statistically significant increase in platform location crossings by 3xTg-AD mice and significantly reduced the latency to find the platform location during the probe test compared to animals treated with an isotype control antibody (Fig. 5c). We observed no significant differences between males and females in the behavioral tests (data not shown). Collectively, these behavioural tests clearly showed that interfering with leukocyte recruitment in the brains of 3xTg-AD mice significantly reduces cognitive impairment at early time points of the disease.

### The α4 integrin-specific antibody reduces neuropathological changes in AD mice

**Figure 6:**
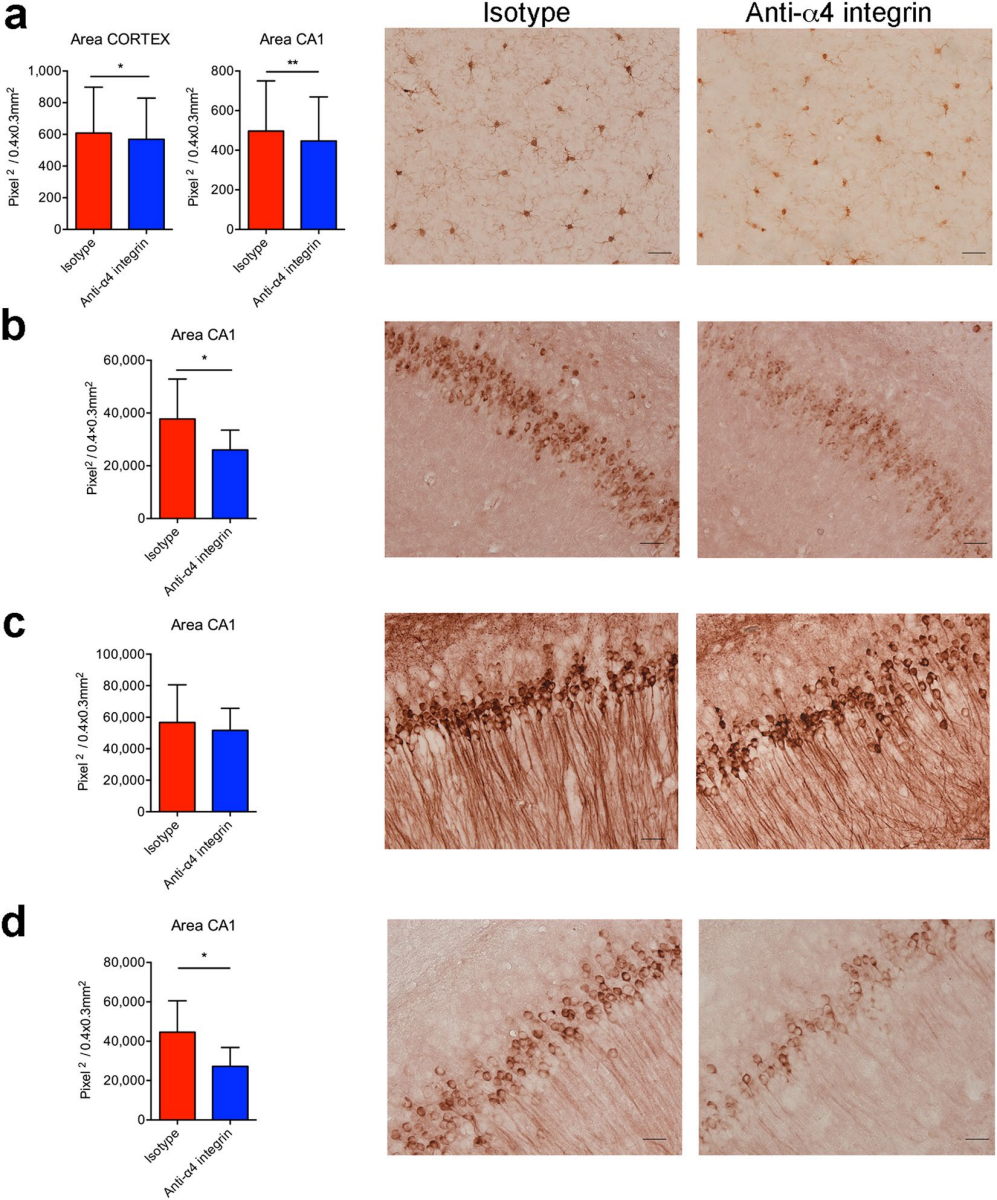
Inhibition of α4 integrins at the early stage of AD reduces microglial activation, Aβ load and hyperphosphorylated tau in 3xTg-AD mice. Mice were treated with the α4 integrin-specific antibody or isotype control at 6 months of age and were left to rest for 1 month before behavioural tests (Fig. 5). Animals were euthanized for neuropathological analysis after the behavioural test at 9–10 months of age. (**a**) Bar graph shows significant differences in microglial activation in the cortex and CA1 hippocampal region of treated mice and isotype controls. Error bars show SD (*P < 0.05, **P < 0.01; Unpaired *t*-test). Representative images show Iba-1 staining of microglia in the cortex of isotype controls (left) and after the α4 integrin blockade (right). (**b**) Quantitative analysis of Aβ accumulation in the CA1 sub-field of hippocampus (left). Error bars show SD (*P < 0.05; Unpaired *t*-test). Representative images show Aβ deposition in the hippocampus of 3xTg-AD mice treated with isotype controls (left) and after the α4 integrin blockade (right). (**c**) Quantitative analysis of total tau protein in the CA1. Representative images show staining for total tau in isotype controls (left) and after the α4 integrin blockade (right). (**d**) Quantitative analysis of the area of AT180^+^ cells in the CA1 of 3xTg-AD mice^.^ Results are shown as mean ± SD (*P < 0.05; unpaired Student’s *t*-test). Representative images show AT180^+^ cells in isotype controls (left) and after the α4 integrin blockade (right). Scale bar in all images = 50 μm. In all panels, n = 3 mice (2 F, 1 M) for the isotype group, and n = 3 mice (2 F, 1 M) for the anti-α4 integrin group.

The beneficial effects of the therapeutic blockade shown by cognitive assessments in 3xTg-AD mice were also supported by neuropathological data (Fig. 6). Mice treated at 6 months of age and tested in behavioural studies as described above were euthanized at 9 months of age and immunohistochemistry experiments were carried out to observe microgliosis, Aβ load and tau pathology.

Microgliosis, as assessed by Iba-1 staining, was less prevalent in mice treated with the α4 integrin-specific antibody than those receiving the isotype control (Fig. 6a). Most microglial cells in the cortex of 3xTg-AD mice treated with the therapeutic blockade had round cell bodies with few ramified processes, indicating a low activation state, whereas microglia in animals treated with the isotype control antibody presented wider and irregular cell bodies with highly ramified, compact and thickened processes indicating a high activation state (Fig. 6a).

The Aβ load was determined by staining with antibody 6E10, revealing less Aβ accumulation in the hippocampal CA1 region of 3xTg-AD mice treated with the therapeutic blockade compared to the isotype control group (Fig. 6b). The accumulation of tau protein in the hippocampus was comparable across all mouse groups (Fig. 6c), but the hyperphosphorylated variant, detected using antibody AT180, was less prevalent in the hippocampal CA1 region in mice treated with the α4 integrin-specific antibody than in those receiving the isotype control (Fig. 6d). We observed no significant differences between males and females in the neuropathology studies (data not shown).

**Figure 7:**
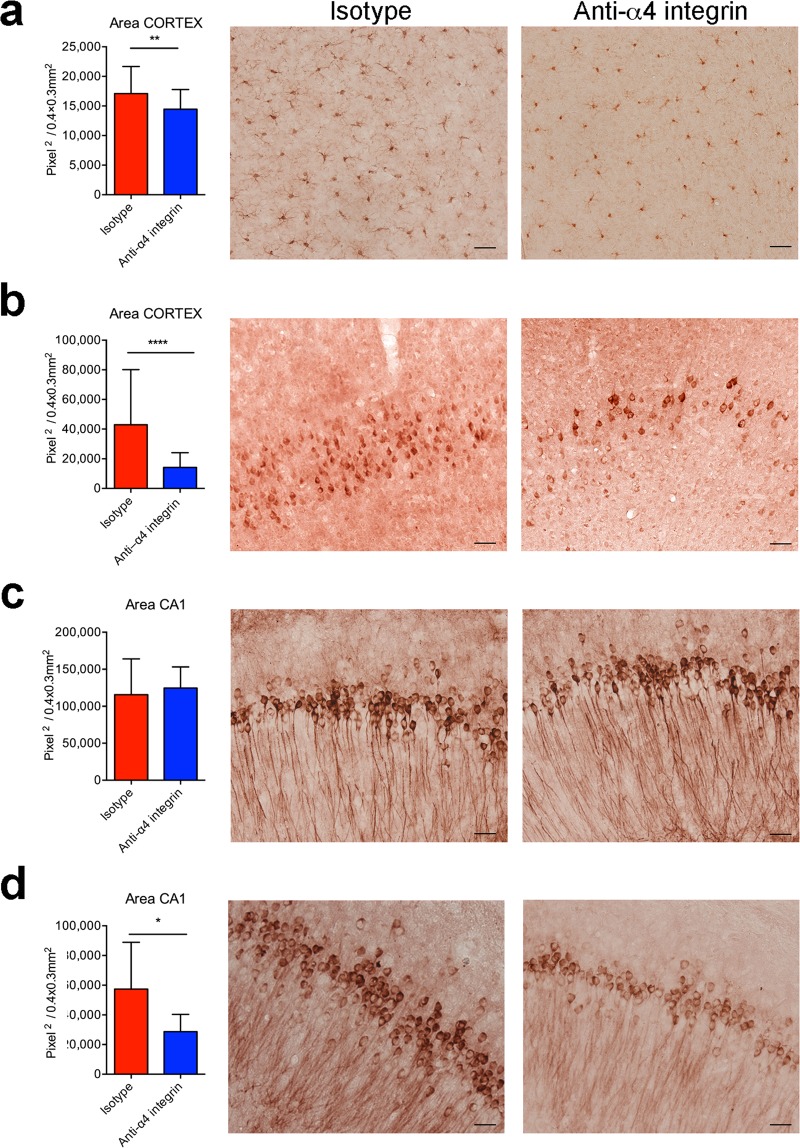
Inhibition of α4 integrins at the late stage of AD reduces microglial activation, Aβ load and hyperphosphorylated tau in 3xTg-AD mice. Mice were treated with the α4 integrin-specific antibody or isotype control at 9 months of age for 4 weeks and were euthanized for neuropathological analysis at 11–12 months of age. (**a**) Bar graph shows significant differences in microglial activation in the cortex and CA1 hippocampal region of treated mice and isotype controls. Error bars show SD (**P < 0.01; Unpaired *t*-test). Representative images show Iba-1 staining of microglia in the cortex of isotype controls (left) and after the α4 integrin blockade (right). (**b**) Quantitative analysis of Aβ accumulation in the CA1 sub-field of hippocampus (left). Error bars show SD (****P < 0.0001; Unpaired *t*-test). Representative images show Aβ deposition in the hippocampus of 3xTg-AD mice treated with isotype controls (left) and after the α4 integrin blockade (right). (**c**) Quantitative analysis of total tau protein in the CA1. Representative images show staining for total tau in isotype controls (left) and the α4 integrin blockade (right). (**d**) Quantitative analysis of the area of AT180^+^ cells in the CA1 of 3xTg-AD mice. Results are shown as mean ± SD (*P < 0.05*;* unpaired Student’s *t*-test). Representative images show AT180^+^ cells in isotype controls (left^)^ and after the α4 integrin blockade (right). Scale bar in all images = 50 μm In all panels, n = 3 mice (2 F, 1 M) for the isotype group and n = 3 mice (2 F, 1 M) for the anti-α4 integrin group.

We also performed neuropathology studies in older mice (9 months old) treated for 4 weeks with the anti-α4 antibody to study the effect of the blockade at a later stage of the disease. Iba-1^+^ cells in the cortex of mice treated with the α4 integrin-specific antibody showed a lower activation state compared to mice treated with the control antibody (Fig. 7a). In addition, less Aβ was detected in the cortex of the treated mice compared to the isotype control group (Fig. 7b). Again, there was no difference between groups in the abundance of total tau protein (Fig. 7c), but the quantity of hyperphosphorylated tau was lower in the group treated with the therapeutic blockade (Fig. 7d). We observed no significant differences between males and females in the neuropathology studies (data not shown). Taken together, our findings demonstrate that the therapeutic targeting of α4 integrins inhibits early disease development and limits the progression of AD-like disease in 3xTg-AD mice.

## Discussion

Leukocyte trafficking is a key process in the development of inflammatory and immune responses. During brain inflammation, the expression of endothelial adhesion molecules increases, and their soluble forms accumulate in the serum, representing biomarkers of endothelial activation and neuroinflammation^13^. We found that neutrophils and T cells extravasated near VCAM-1^+^ cerebral vessels in 3xTg-AD mice, suggesting a role for this endothelial adhesion molecule in leukocyte migration into the brain during AD. These results are supported by our previous data showing that VCAM-1 is upregulated in the brains of AD-like models compared to wild-type control mice^7^. Although VCAM-1 expression in the cerebral vessels of human AD patients has not been investigated, higher levels of soluble VCAM-1 are found in plasma samples from AD patients compared to controls, and the levels of soluble VCAM-1 correlate with more advanced dementia and changes in white matter hyper-intensity^22,23^. This indicates that VCAM-1 is a candidate biomarker for vascular inflammation associated with and potentially contributing to cognitive decline during AD.

The main leukocyte ligand of endothelial VCAM-1 is α4β1 integrin, a key mediator of leukocyte adhesion on activated endothelial cells during inflammation^13,14^. Our EVIM analysis in 3xTg-AD mice revealed that blocking the α4 chain inhibits rolling interactions in cortical venules, suggesting that VLA-4 contributes to the leukocyte–vascular interactions in AD. Previously, α4β1 was shown to mediate the trafficking of activated T cells to sites of inflammation, including the brain in chronic inflammatory diseases such as multiple sclerosis^13,24^. The presence of invasive T-cell subpopulations in the brain has been previously been shown in human AD patients and animal models of AD^6,8–12^. Although their role in disease progression remains unclear, both CD4^+^ and CD8^+^ T cells were found to adhere to the vascular endothelium or migrate into the parenchyma of AD patients and animal models^6,8–12,25,26^. The number of infiltrating T cells was higher in AD patients than control subjects, especially in the hippocampus and other limbic structures^6,8–10^. Interestingly, mild cognitive impairment (MCI) and mild forms of AD are associated with a greater number of activated CD4^+^ and CD8^+^ T cells in the cerebrospinal fluid (CSF), supporting a role for the migration of activated T cells into the brain during AD^27^. Our data revealed a progressive age-dependent increase in the proportion of circulating CD4^+^ T cells expressing α4 integrin together with a higher MFI representing α4 integrin expression on these cells. Moreover, we observed an increase in the abundance of CD4^+^ T cells migrating into the brains of 3xTg-AD mice compared to wild-type animals. Together, these data suggest that CD4^+^ T cells are more activated in AD mice and that α4 integrins may have a role in CD4^+^ T cell extravasation in the brains of 3xTg-AD mice. We also found that α4 integrin was expressed on circulating CD8^+^ T cells, but there was no difference between 3xTg-AD and wild-type mice. However, our previous studies showed that chronic brain inflammation is associated with CD8^+^ T cells preferentially using mucin PSGL-1, rather than VLA-4, for rolling interactions, suggesting this may be the case also in AD^21^.

The α4 integrin chain was also expressed on a proportion of peripheral neutrophils in 3xTg-AD mice at 6 and 9 months of age, but we observed no difference in α4 expression on circulating neutrophils in the brains of 3xTg-AD and wild-type mice, suggesting that VLA-4 is not required for neutrophil trafficking in the AD brain. Therese results are in line with our previous data showing that integrin LFA-1 is the primary adhesion molecule responsible for the intravascular adhesion and intraparenchymal migration of neutrophils in the brains of 3xTg-AD mice^7^. Previous studies have shown that blood neutrophils express low levels of VLA-4 compared to bone marrow neutrophils, suggesting that VLA-4 integrin expression declines during neutrophil maturation^28,29^. However, interactions between VLA-4 and VCAM-1 may provide an alternative pathway for neutrophil migration during inflammatory responses, as previous studies have shown that VLA-4 is rapidly upregulated on neutrophils following activation and during transmigration through the endothelial wall^18^. Several studies have shown that VLA-4 mediates adhesive interactions on the endothelium during various inflammatory conditions^30–35^. Furthermore, our previous research has shown that α4 integrins can mediate neutrophil rolling in the inflamed brain microcirculation following status epilepticus, suggesting the interaction between VLA4 and VCAM-1 may is also act as a neutrophil recruitment pathway during sterile brain inflammation^36^. Indeed, neutrophil adhesion mediated by VLA-4 in brain vessels also has a key role in stroke models, and the blockade of α4 integrins effectively protected mice from stroke-associated behavioural impairment^35^. Previous studies have shown that VLA-4 can also mediate the tethering and rolling of eosinophils and monocytes, thus we cannot exclude the possibility that the anti-α4 treatment interfered with the recruitment of myeloid cells into the brain of 3xTg-AD mice^16,17,19^. VLA-4 may also bind fibronectin, which has been shown to accumulate in the brain of AD patients, so it is possible that the beneficial effect of blocking α4 integrin may also partially reflect the inhibition of adhesive interactions between these molecules^19,37–39^.

In agreement with our data showing the increased activation of circulating CD4^+^ T cell helper lymphocytes in 3xTg-AD mice, recent studies have shown these animals have higher serum levels of IL-2, TNFα, IL-17 and GM-CSF at 9 months of age compared with controls, suggesting the increased activation of CD4^+^ T cells with Th1 and Th17 phenotypes in 3xTg-AD mice^40^. The concentration of GM-CSF and IL-12, two cytokines associated with the Th1 immune response, is higher in the brains of 3xTg-AD, along with higher concentrations of IL-5 and the chemokine CCL2^41^. It is well documented that a VLA-4 blockade targets Th1 cells, leading to a reduction in the levels of IFNγ, TNFα/β and GM-CSF at the inflammation site, including the CNS during experimental autoimmune encephalomyelitis, the animal model of multiple sclerosis (MS)^42–45^. Interestingly, the treatment of 3xTg-AD mice with intravenous immunoglobulin (IVIg) reduces the accumulation of IL-12 and Th2 response/atopy-related IL-5 in the brain, but has no effect on GM-CSF levels, suggesting that anti-α4 treatment and IVIg therapy may have different effects on cytokine production.

Blocking the α4 integrin chain has anti-inflammatory effects in animal models of atherosclerosis, autoimmune diseases and asthma^42^. Similarly, blocking VLA-4 has a beneficial effect on brain inflammation in animal models of multiple sclerosis, epilepsy and stroke^24,35,36,43^. Here, we found that a therapeutic blockade of α4 integrins improves the memory in cognitive tests, suggesting this therapeutic approach interferes with disease progression and cognitive impairment. We also found that the blockade reduced the neuropathological hallmarks of AD, including Aβ accumulation and tau hyperphosphorylation, further supporting the beneficial effect of drugs targeting α4 integrin. Recent data showing a correlation between tau pathology and T cell accumulation in post-mortem brain samples of AD patients support our data suggesting a role for T cell migration in AD pathogenesis^12^. Prolonged microglial activation may have a detrimental role in AD, and here we show that the blockade of α4 integrin inhibits this process in 3xTg-AD mice, highlighting the anti-inflammatory effect of anti-adhesion therapy. Our results agree with recent neuropathology studies in *APP/PS1* mice, a model of brain amyloidosis, showing that treatment with an anti-CD49d antibody reduces microgliosis, astrogliosis, CD4 immunoreactivity and synaptic changes, without altering the Aβ plaque load^46^. However, we observed lower levels of intracellular Aβ accumulation during earlier stages of AD and determined how blocking α4 integrins affects cognitive functions using a more complex model, in which mice develop both amyloid and tau pathologies, thus more closely representing AD neuropathology in humans. We found that the therapeutic α4 integrin blockade clearly inhibited leukocyte–vascular interactions, indicating that anti-adhesion therapies may have a beneficial effect in AD. The blockade of LFA-1 integrin has also been shown to reduce memory decline and the neuropathological hallmarks of AD in transgenic mouse models, further supporting anti-integrin drugs as a new therapeutic approach in AD^7^.

The α4 integrin subunit can pair with either the β1 or β7 subunits, so drugs targeting α4 integrin not only inhibit α4β1/VCAM-1 interactions but also α4β7/MAdCAM-1 binding^47,48^. Blocking the α4 chain of VLA-4 with natalizumab, a humanized monoclonal antibody, has a therapeutic effect in patients with multiple sclerosis and Chron’s disease^49,50^. Following initial promising results, natalizumab was approved in 2004 by the US Food and Drug Administration and the European Medicines Agency as monotherapy for highly active relapsing–remitting  multiple sclerosis. The observational study on multiple sclerosis patients treated with natalizumab for up to 2 years demonstrated highly improved cognitive performance and a significant reduction of clinical symptoms, suggesting that the efficacy of natalizumab against neuroinflammation and neurodegeneration may be also useful in AD^51^. Our previous work showed that natalizumab also reduces the frequency and severity of seizures in a patient with multiple sclerosis and epilepsy, further supporting anti-leukocyte adhesion therapy as a promising therapeutic approach in patients with brain inflammation^52^. However, natalizumab and other anti-integrin or immunosuppressive therapies can induce the viral brain disease progressive multifocal leukoencephalopathy (PML) in patients with autoimmune disorders, especially in immunosuppressed subjects^53^. The fact that AD patients do not normally receive immunosuppressive therapy, and availability of improved tests to detect patients at risk of PML argues in favour of α4-targeted therapy in AD^53^. Interestingly, an association between two polymorphisms (−269 C/A and +3061 A/G) in the *ITGA4* gene (encoding α4 integrin) and the risk of AD has been reported recently, providing further evidence that VLA-4 contributes to AD pathogenesis^54^. The *ITGA4* +3061AG genotype is also associated with the development of multiple sclerosis, which is thought to be mediated by T cells^13,15,24,55^. This suggests that the AG variant may change the VLA-4 α4 subunit conformation, making it bind with higher affinity to its ligand VCAM-1, increasing the accumulation of T cells in chronic inflammatory diseases of the CNS, such as multiple sclerosis and AD. However, further studies are needed to understand the functional implications of *ITGA4* gene polymorphisms on leukocyte trafficking in the brain during chronic neuro-inflammatory diseases.

In conclusion, our data show that α4 integrins control leukocyte–endothelial interactions and that a therapeutic blockade can significantly inhibit neuropathological hallmarks such as Aβ deposition and tau hyperphosphorylation, as well as reducing memory decline in a 3xTg-AD model. Drugs that block VLA-4 may therefore represent a new therapeutic approach in AD, which can be rapidly translated to the clinic.

## Methods

### Ethics statement

The research involving animals was authorized by the Italian Ministry of Health, Department of Veterinary Public Health, Nutrition and Food Safety, Directorate General of Animal Health and Veterinary Medicine, as required by Italian legislation (D. Lgs 26/2014, application of European Directive 2010/63/EU). All efforts were made to minimize the number of animals used and their suffering during the experimental procedures. All mouse experiments were carried out in accordance with guidelines prescribed by the Ethics Committee for the use of laboratory animals for research purposes at the University of Verona and by the Italian Ministry of Health.

### Mice

The 3xTg-AD mice (MMRRC stock no. 34830-JAX) and wild-type control B6129SF2/J (stock no. 101045) were purchased from The Jackson Laboratory. The 3xTg-AD mouse expresses the human mutant APPswe and PS1M146V mutant alleles associated with familial AD and the TauP301L mutation associated with human tau pathology, and therefore develops both amyloid and tau pathologies. Animals were housed in pathogen-free climate-controlled facilities and were provided with food and water ad libitum.

### Isolation of peripheral leukocytes and analysis by flow cytometry

Blood samples were collected from the retro-orbital plexus of anesthetized mice in sodium heparinized capillaries at a 50:50 ratio of blood to 1% dextran plus 10 U/ml sodium heparin. After erythrocyte sedimentation, the overlying supernatant plasma-dextran suspension of leukocytes was washed in phosphate-buffered saline (PBS), followed by erythrocyte lysis. The leukocytes were then labeled with the following anti-mouse antibodies: anti-CD45-Vioblue (clone REA737, Miltenyi Biotec), anti-CD11b-APC-Cy7 (clone M1/70, Biolegend), anti-Ly6G-FITC (clone REA526, Miltenyi), anti-CD8α-PE-Cy7 (clone 53-6.7, Biolegend), anti-CD4-APC (clone RM4-4, Biolegend), cd49d-PE (clone R1-2, Biolegend). Cell viability was detected using 7AAD (eBioscience). Specimens were acquired by flow cytometry using a MACSQuant Analyzer (Miltenyi Biotec). Data were analyzed using FlowJo software.

### Immunofluorescence staining

To stain cerebral blood vessels, 100 μg of Texas Red tomato lectin (Vector) in PBS was injected i.v. through the tail vein of 3xTg-AD and wild-type control mice. After 15 min, mice were perfused intracardially with PBS followed by 4% paraformaldehyde. Brains were collected, fixed overnight in 4% paraformaldehyde, rinsed and cryoprotected. Sections were prepared from the anterior hippocampus through the bregma – 2.9 mm at an intersection interval of 500 μm (every fourth section). Free-floating coronal sections (30 μm) were incubated in blocking solution (2% normal goat serum and 0.4% Triton X-100) for 1 h at room temperature and then with primary antibodies for 18 h at 4 °C (Alexa 647 anti-VCAM1 (clone 429, MVCAM.A from Biolegend), Alexa-488 anti-CD3 and Alexa-647 anti-CD3 (clone 17A2, Biolegend), Alexa 488 anti-CD4 (clone GK 1.5, Biolegend). After rinsing in PBS, the nuclei were stained with DAPI for 8 min in the dark, and the sections were transferred to glass slides and mounted with Dako medium. Images were acquired with Axio Imager Z2 (Zeiss, Germany).

### EIVM using a digital camera system

#### Surgical preparation

Mice were anesthetized and the core body temperature was monitored and maintained using a regulated heating pad. Hair on the scalp was removed with an electric razor. The scalp was then sterilized with alcohol. An incision was made along the midline of the scalp to expose the skull overlying the cortical region of interest. A 1-mm diameter region of skull was thinned using a high-speed micro drill and a stainless-steel burr. Drilling was paused every few seconds to prevent heating, and bone dust was removed using a compressed air canister. Care was taken not to deflect the skull during drilling. Animals (n = 2) showing signs of damage, such as subdural or epidermal bleeding, with the potential to interfere with imaging and data analysis, were excluded from the study. Mice were given a bolus of warm saline for rehydration and were allowed to recover from anesthesia on a water-circulating heating pad. A 24 × 24 mm coverslip was applied to the scalp. A round camera (11 mm internal diameter) was attached on the coverslip and filled with water^56^. To analyze total leukocyte interactions by epifluorescence microscopy, endogenous leukocytes were stained with 100 µl of 1 mg/ml Rhodamine 6G (Sigma Aldrich) by i.v. injection into the lateral tail vein^57^.

#### Image acquisition

Anesthetized mice were placed on a customized upright TCS SP5 AOBS microscope (Leica Microsystems) equipped with an Olympus XLUMPlanFI 20x objective (water immersed, NA 0.95) using confocal settings, and a heat pad was used to maintain the body temperature. A monochromatic DFC360 FX digital camera was used to capture the images (Leica Microsystems). Rhodamine 6G-labeled endogenous leukocyte interactions were recorded on a single focal plane by capturing sequential images at intervals of 0.4 s using the Leica acquisition software.

#### Image analysis

The image stacks obtained with the digital camera were transformed into time-lapse sequences at intervals of 0.4 sec (2.5 fps) using Imaris software (Bitplane), for the analysis of cell interactions. Post-capillary venules (diameter 30–70 µm) were chosen for observation. The velocities of 20 consecutive freely-flowing cells per venule were manually calculated, and the velocity of the fastest cell in each venule (Vfast) was used to calculate the mean blood flow velocity (Vm) according to Eq. (1):

1 $${{\rm{V}}}_{{\rm{m}}}={{\rm{V}}}_{{\rm{fast}}}/(2-{{\rm{\varepsilon }}}^{2})$$

where ε is the ratio of the leukocyte diameter to the vessel diameter. Leukocytes were considered to interact if they traveled at velocities below the critical velocity (Vcrit) which was determined using Eq. (2)^58^:

2 $${{\rm{V}}}_{{\rm{crit}}}={{\rm{V}}}_{{\rm{m}}}\times {\rm{\varepsilon }}\times (2-{\rm{\varepsilon }})$$

Cell tracking in time-lapse movies was carried out automatically using Imaris software. Briefly, the bi-dimensional spatial position of each cell in the post-capillary venules was detected based on the centroid fluorescence intensity. Cells were tracked over time, choosing different minutes at random in each acquisition.

### EIVM using an analog camera system

#### Surgical preparation

We performed epifluorescence microscopy in real time using an Olympus BX50WI microscope to study rolling and adhesive interactions in detail. Mice were anesthetized and the skull overlying the cortical region of interest was exposed as described above. The top of chamber was filled with sterile saline and a 24 × 24 mm coverslip was applied and fixed with silicon grease. A round camera was attached^56^ and endogenous leukocytes were stained with Rhodamine 6G as described above.

#### Image acquisition

Anesthetized mice were placed on an upright Olympus BX50WI microscope equipped with a water immersion objective with a long focal distance (Olympus Achroplan, focal distance 3.3 mm, NA 0.5). The images were visualized on a Sony SSM-125CE monitor using a VE-1000 SIT analog monochromatic silicon-intensified target camera (Dage-MTI). Real-time recordings were digitized and stored using an HDV DN-400 recorder (Datavideo Technologies Co.).

#### Image analysis

Digital video files from the VE-1000 SIT analog camera were manually analyzed at intervals of 0.04 s (25 fps). Rolling leukocytes were defined as cells moving at a markedly lower velocity than surrounding erythrocytes inside a given vessel. Rolling cells passing through a vessel over the course of 1 min were tracked manually. Leukocytes that remained stationary on the venule wall for ≥30 s were considered adherent. One-minute time windows were chosen randomly for analysis from each acquisition, and data from three vessels (from each mouse) were averaged^59^.

### The α4 integrin blockade for EIVM

To determine how α4 integrin influences the interactions between endogenous leukocytes and the inflamed endothelium, we administered 500 μg of the α4 integrin-specific antibody (PS/2 clone) to 3xTg-AD mice in a single i.v injection into the lateral tail vein. Leukocytes interactions were acquired before and after treatment, with the before-treatment interactions used as a negative control. We also administered a second group of mice with an anti-RAS antibody (Y13259 clone) as an isotype control. Antibodies were diluted to 1 mg/ml in sterile endotoxin-free PBS.

### Treatment of mice

The mice were housed in groups of 2–4 same-sex littermates and individually, identified by ear punch. Mice were injected i.p. with 500 μg of each antibody for the first treatment, then with 300 μg every other day (anti-mouse CD49d, clone PS/2 from Bioxcell). Treatment was continued for 4 weeks and then mice were left to rest for one month until behavioural testing. The tests were ordered from least to most stressful to reduce the effect of stress on learning and memory, so the Y maze test was performed first, then the Morris water maze (MWM) test and finally contextual fear conditioning. Mice were euthanized after behavioural assessment. The perfused brains were fixed in 4% PFA, embedded in OCT (DDK Italia) and stored at −80 °C.

### Spontaneous alternation Y-maze test

This test assesses hippocampus-dependent spatial learning memory. Each mouse was placed in the centre of a symmetrical Y-maze and was allowed to explore freely during an 8-min session. The sequence and total number of arms entered were recorded. Arm entry was scored when the mouse placed all paws completely inside the arm. Alternation was defined as successive entries into the three arms in overlapping triple sets. The alternation percentage was calculated as the number of triads containing entries into all three arms divided by the maximum possible number of alternations (the total number of arms entered − 1) × 100. To diminish odour cues, the maze was cleaned with 70% ethanol. Experiments were blinded with respect to the genotype of the mice and the treatment.

### Morris water maze test

Spatial reference memory was assessed using the MWM test with modifications, as previously described^60^. The apparatus was an open circular white pool 1.5 m diameter and 0.6 m high (Ugo Basile) filled with water (25 ± 1 °C) to a height of 30 cm and made opaque with starch. The track of mice was recorded using a camera positioned 2 m above the centre of the pool, and connected the ANY-maze software video tracking system (Stoelting). The maze was designed with two virtual principal axes, with each line bisecting the maze perpendicular to the other to divide the maze into four equal quadrants. The end of each line demarcated four cardinal points: north, south, east, and west. Mice were given four trials per day, each lasting 90 s, for four consecutive days, to find the hidden platform (10 cm in diameter) submerged about 1 cm below the opaque water surface and located at a fixed quadrant centre. The platform remained in the same position throughout the learning trials. The starting position was a randomly chosen quadrant of the pool. If the mouse failed to reach the escape platform within the given time, the test was terminated and the mouse was gently guided to the platform and allowed to remain for 10 s. Then, the mouse was dried and returned to its cage until the next trial. Retention of the spatial training was assessed 24 h after the last trial. In the probe trial, the platform was removed and the mice were given 60 s to search the target quadrant. The escape latencies (s), the number of platform crossing in target quadrant, and the swimming speed (cm/s) were recorded and analysed using ANY-maze software.

### Contextual fear conditioning test

Contextual fear conditioning was performed in 30 × 24 × 21 cm operant chambers (Ugo Basile). Each chamber was equipped with a stainless-steel rod floor through which a footshock could be administered, two stimulus lights, one house light, and a solenoid, all controlled using ANY-maze computer software. Mice were trained and tested on two consecutive days. Training consisted of placing a mouse in a chamber, providing an illuminating stimulus and house lights, and allowing exploration for 2 min. A 15-s tone stimulus (2 Hz) then co-terminated with 2-s foot shock (1.5 mA). The pairing of stimuli was repeated twice, at 2-min intervals. Thirty seconds after the second shock, the mice were removed from the chamber. After a further 20 h, mice were placed back into the same training chamber for the contextual test for 5 min, with no tones or shocks delivered, where freezing behaviour was recorded by the experimenter. Freezing was defined as lack of movement except that required for respiration. At the end of the contextual test, mice were returned to their home cage. Approximately 2 h later, mice were placed in a novel environment for a cued fear memory test. The new environment consisted of a coloured Plexiglas sheet that covered the steel rods of the floor, black and white striped plastic on the walls of the chamber, and the introduction into the testing chamber of a novel odour. After 2.5 min without any stimulus, mice were exposed to the auditory cue for the remaining minutes, and freezing behaviour was again scored. The freezing score was expressed as a percentage for each portion of the test. Memory for the context for each subject was obtained by subtracting the percentage of freezing in the novel environment from that in the contextual environment. Experiments were blinded with respect to the genotype of the mice and the treatment.

### Histopathological analysis

Mouse brains were cut into 30-μm coronal sections, immersed in blocking solution (2% normal goat serum and 0.4% Triton X-100) and then incubated for 18 h at 4 °C with the following primary antibodies: anti-mouse ionized calcium-binding adaptor molecule-1 antibody (Iba-1) (Wako); anti-human Aβ (clone 6E10) (Covance); anti-human total tau antibody (clone HT7) and anti-human phospho-tau (Thr231) antibody (clone AT180 from Thermo Fisher Scientific). Staining required epitope retrieval using 70% formic acid for 20 min for Aβ and 10 mM sodium citrate buffer (pH 8.5) pre-heated to 85 °C in water bath for tau. After washing with 0.05% Tween-20 in PBS, we added 3% H_2_O_2_ for 10 min at room temperature before washing the sections and incubating them with the biotinylated secondary antibody (goat anti-rabbit and goat anti-mouse antibodies). Immunoreactivity was visualized using the VECTASTAIN ABC kit (Vector) for 30 min and Vector NovaRED (Vector) as the chromogen for 3 min at room temperature. Finally, brain sections were washed with distilled water, transferred to glass slides, dehydrated in 95% and 100% ethanol for 1 min each and mounted with Eukitt mounting medium (Sigma-Aldrich). Images were acquired blindly with respect to the genotype and the treatment using an Axio Imager Z2 fluorescence microscope (Zeiss).

### Quantification of microglia, Aβ load and tau pathology

The areas covered by Iba-1^+^ microglia, Aβ deposits, total tau and phospho-tau positive neurons were determined in coronal sections throughout the cortex and the hippocampus. Sections were taken from the anterior hippocampus through the bregma – 2.9 mm at an intersection interval of 500 μm (every fourth section) – in order to analyze the whole area of the cortex and the hippocampus. The sections were analyzed blindly with respect to the genotype and treatment using ImageJ v1.32j software.

### Statistical analysis

All data were collated in Prism 6 (GraphPad Software) which was also used for statistical analysis. Where indicated, statistical analysis was performed using an unpaired two-tailed *t*-test, multiple t tests or one-way and two-way ANOVA. All data are presented as means ± standard deviation (SD). P < 0.05 was considered as statistically significant. Cohen’s d statistic and magnitude were measured by R version 3.6.1 (2019-07-05) for each study (Supplementary Table 1). Age and sex-matched 3xTg-AD and wild-type control mice were randomly assigned to each treatment. Data collection from flow cytometry experiments, behavioural tests and immunohistological analysis was performed in a blinded fashion.

## Supplementary information

Supplementary information
