# Supplementary material for: Blockade of α4 integrins reduces leukocyte–endothelial interactions in cerebral vessels and improves memory in a mouse model of Alzheimer’s disease
Source: Sci Rep. 2019 Aug 19;9:12055. doi: 10.1038/s41598-019-48538-x (PMC6700124; doi:10.1038/s41598-019-48538-x)
Supplement: Supplementary file 1 — Supplementary information [file 41598_2019_48538_MOESM1_ESM.pdf]

Supplementary Information

Blockade of α4 integrins reduces leukocyte–endothelial interactions in cerebral vessels and improves memory in a mouse model of Alzheimer's disease

Enrica Pietronigro[1*], Elena Zenaro[1*], Vittorina Della Bianca[1], Silvia Dusi[1], Eleonora Terrabuio[1], Giulia Iannoto[1], Anna Slanzi[1], Somayehsadat Ghasemi[1], Rajasekar Nagarajan[1], Gennj Piacentino[1], Gabriele Tosadori[1,2], Barbara Rossi[1], Gabriela Constantin[1, 2, 3]

[1]Department of Medicine, University of Verona, 37134 Verona, Italy

[2]The Center for Biomedical Computing (CBMC), University of Verona, 37134 Verona, Italy

[*]These authors equally contributed to this work

[3]Address correspondence to: Prof. Gabriela Constantin, Department of Medicine, Section of General Pathology, University of Verona, Strada le Grazie 8, Verona 37134, Italy; Phone: +39-045-8027102; Email: gabriela.constantin@univr.it

Running title: Role of α_4 integrins in Alzheimer's disease

Keywords: leukocyte trafficking, Alzheimer's disease, α*4 integrins, in vivo imaging, therapeutic approaches*

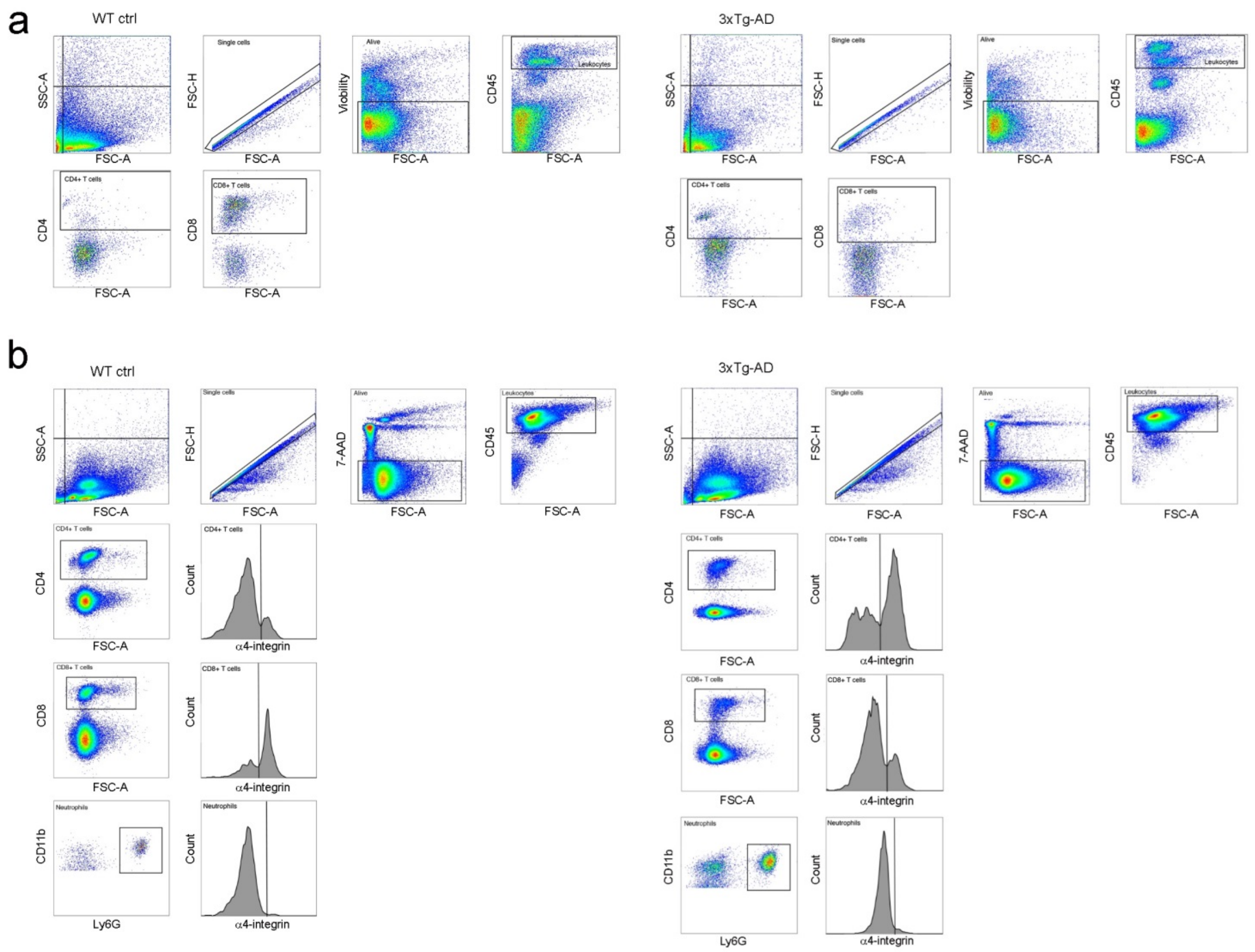

Supplementary Figure 1. Gating strategies for flow cytometry experiments.

Representative gating strategy used during FACS analysis for the identification of (**a**) brain-infiltrating CD45$^+$ cells and (**b**) peripheral leukocytes in wild-type control (WT ctrl) and 3xTg-AD mice by simultaneous labeling with antibodies recognizing lymphocyte and neutrophil markers. Doublets were removed from the analysis by the sub-gate identification of singlets using FSC-A and FSC-H. **a**) Dead cells were removed from the analysis using Viobility 405/520 fixable dye. CD45^{high} cells were sub-gated using FSC-A plus a CD4 double gate to define CD4$^+$ T cells, and FSC-A plus a CD8 double gate to define CD8$^+$ T cells. **b**) Dead cells were removed from the analysis using 7AAD. CD4$^+$ and CD8$^+$ T cells were identified by gating on CD45$^+$ cells, whereas Ly6G$^+$ neutrophils were detected by gating CD11b/Ly6G on CD45$^+$ leukocytes. The α4 integrin expression in each population (CD8$^+$ T cells, CD4$^+$ T cells and Ly6G$^+$ neutrophils) is shown in the histograms.

Supplementary Table I. Effect size statistics

FACS analysis (WT ctrl vs 3xTg-AD)	Cohen's *d*	Magnitude
**Brain**		
% of $CD4^+$ cells on $CD45^{high}$ (6 months)	-0.3924413	Small
% of $CD4^+$ cells on $CD45^{high}$ (9 months)	-1.938403	Large
% of $CD8^+$ cells on $CD45^{high}$ (6 months)	-0.05060928	Negligible
% of $CD8^+$ cells on $CD45^{high}$ (9 months)	2.790802	Large
**Blood**		
% α4 $integrin^+$ $CD4^+$ cells (6 months)	-2.75399	Large
% α4 $integrin^+$ $CD4^+$ cells (9 months)	-2.195376	Large
MFI α4 $integrin^+$ expression on $CD4^+$ cells (6 months)	0.2582844	Small
MFI α4 $integrin^+$ expression on $CD4^+$ cells (9 months)	-6.414628	Large
% α4 $integrin^+$ $CD8^+$ cells (6 months)	1.356608	Large
% α4 $integrin^+$ $CD8^+$ cells (9 months)	1.175441	Large
MFI α4 $integrin^+$ expression on $CD8^+$ cells (6 months)	0.6933269	Medium
MFI α4 $integrin^+$ expression on $CD8^+$ cells (9 months)	0.3108755	Small
% α4 $integrin^+$ $Ly6G^+$ cells (6 months)	0.1284168	Negligible
% α4 $integrin^+$ $Ly6G^+$ cells (9 months)	-0.1840141	Negligible
MFI α4 $integrin^+$ expression on $Ly6G^+$ cells (6 months)	0.9542835	Large
MFI α4 $integrin^+$ expression on $Ly6G^+$ cells (9 months)	1.210988	Large

IVM studies	Cohen's *d*	Magnitude
**Digital**		
Number of interacting cells ≤ Vcrit (WT ctrl vs 3xTg-AD)	-2.835166	Large
% of interacting cells ≤ Vcrit (Untreated vs Anti-α4)	1.582386	Large
% of interacting cells ≤ Vcrit (Untreated vs Isotype)	-0.2805429	Small
**Analog**		
% of leukocyte rolling (Untreated vs Anti-α4)	1.283774	Large
% of leukocyte rolling (Untreated vs Isotype)	0.5024471	Medium

Behavioral tests	Cohen's d	Magnitude
**Y maze**		
% Alternation (WT ctrl vs 3xTg-AD	1.507574	Large

Isotype)		
% Alternation (3xTg-AD Isotype vs 3xTg-AD Anti-α4)	-1.209133	Large
**CFC**		
% Freezing (WT ctrl vs 3xTg-AD Isotype)	1.343395	Large
% Freezing (3xTg-AD Isotype vs 3xTg-AD Anti-α4)	-1.058881	Large
**MWM**		
Escape latency (WT ctrl vs 3xTg-AD Isotype)	0.7383913	Medium
Escape latency (3xTg-AD Isotype vs 3xTg-AD Anti-α4)	1.307888	Large
Platform location crossing (WT ctrl vs 3xTg-AD Isotype)	1.487006	Large
Platform location crossing (3xTg-AD Isotype vs 3xTg-AD Anti-α4)	-1.553772	Large
Latency to cross platform location (WT ctrl vs 3xTg-AD Isotype)	-2.243153	Large
Latency to cross platform location (3xTg-AD Isotype vs 3xTg-AD Anti-α4)	1.247004	Large

**Neuropathology (Isotype vs Anti-α4)**	**Cohen's *d***	**Magnitude**
**Treatment at 6 months**		
Area Cortex Iba-1	0.1447865	Negligible
Area CA1 Iba-1	0.2103128	Small
Area CA1 6E10	0.9596423	Large
Area CA1 HT7	0.2634473	Small
Area CA1 AT180	1.321709	Large
**Treatment at 9 months**		
Area Cortex Iba-1	0.647467	Medium
Area Cortex 6E10	0.9800084	Large
Area CA1 HT7	-0.2208119	Small
Area CA1 AT180	1.408082	Large

The magnitude was assessed using the thresholds provided by Cohen (1992)[1], i.e. $d < 0.2$ negligible, $d < 0.5$ small, $d < 0.8$ medium, otherwise large

Supplementary Methods

Isolation of brain leukocytes and flow-cytometry analysis

Mice were anesthetized and perfused through the left cardiac ventricle by injecting cold PBS. The brain was digested with 20 U/ml of DNaseI and 1 mg/ml collagenase at 37°C for 45 min. Cells were isolated by passing the digested tissue through a 70-μm cell strainer. They were then resuspended in 30% Percoll and loaded onto 70% Percoll. The tubes were centrifuged at 1300 × g for 20 min at 4°C. Cells were removed from the interphase, washed and labeled with the following anti-mouse antibodies: anti-CD45-PE, anti-CD11b-PE-Vio770, anti-Ly6G-APC-Vio770, anti-CD3 PerCP-Vio700, anti-CD8a VioBright-FITC, and anti-CD4 VioBlue. Cells were acquired by flow cytometry with MACSQuant Analyzer (Miltenyi Biotec). Data were analyzed using FlowJo software.

Supplementary Reference

1. Cohen, J. A power primer. *Psychol Bull*. **112,** 155-159 (1992).
